# Rapid decline of noninvasive fibrosis index values in patients with hepatitis C receiving treatment with direct-acting antiviral agents

**DOI:** 10.1186/s12876-019-0973-5

**Published:** 2019-04-27

**Authors:** Wei-Fan Hsu, Hsueh-Chou Lai, Wen-Pang Su, Chia-Hsin Lin, Po-Heng Chuang, Sheng-Hung Chen, Hung-Yao Chen, Hung-Wei Wang, Guan-Tarn Huang, Cheng-Yuan Peng

**Affiliations:** 1 Division of Hepatogastroenterology, Department of Internal Medicine, China Medical University Hospital, No. 2, Yuh-Der Road, 40447 Taichung, Taiwan; 2 Graduate Institute of Biomedical Science, China Medical University, 40442 Taichung, Taiwan; 3 School of Chinese Medicine, China Medical University, 40442 Taichung, Taiwan; 4 School of Medicine, China Medical University, 40442 Taichung, Taiwan

**Keywords:** Aspartate aminotransferase/platelet ratio index (APRI), Chronic hepatitis C, Direct-acting antiviral agents, FIB-4, Liver stiffness measurement, Noninvasive fibrosis index, Platelet

## Abstract

**Background:**

Studies on temporal changes in noninvasive fibrosis indices and liver stiffness measurement (LSM) in patients with chronic hepatitis C (CHC) treated with direct-acting antiviral agents (DAAs) are limited.

**Methods:**

We retrospectively enrolled consecutive patients with CHC who had received DAAs.

**Results:**

In total, we recruited 395 consecutive patients, of which 388 (98.2%) achieved a sustained virologic response (SVR) at 12 weeks after therapy. In patients who received DAA therapy and achieved SVR 12 weeks after therapy (*n* = 388), the median aspartate aminotransferase/platelet ratio index (APRI) value decreased from 1.19 (0.62–2.44) at baseline to 0.50 (0.32–0.95), 0.51 (0.31–0.92), 0.48 (0.31–0.88), and 0.52 (0.33–0.92) at week 2, week 4, end of therapy, and PW12, respectively (all *P* < 0.001). The median FIB-4 value decreased from 2.88 (1.56–5.60) at baseline to 2.10 (1.30–3.65), 2.15 (1.30–3.65), 2.11 (1.37–3.76), and 2.22 (1.45–3.82) at week 2, week 4, end of therapy, and PW12, respectively (all *P* < 0.001). The median alanine aminotransferase level significantly decreased from week 2 until PW12 (all *P* < 0.001). The platelet count significantly increased from 2 weeks after DAA therapy initiation until PW12 (all *P* < 0.001); however, the magnitude of changes in the platelet count was low. In patients with paired LSMs obtained using acoustic radiation force impulse elastography at baseline and PW12 (*n* = 199), the median LSM decreased from 1.78 (1.25–2.30) m/s at baseline to 1.38 (1.14–1.88) m/s at PW12 (*P* < 0.001).

**Conclusions:**

Noninvasive fibrosis indices, namely APRI and FIB-4, exhibited a rapid and sustained decline from week 2 until PW12 in patients with CHC who achieved SVR to DAA therapy. The rapid decline in APRI and FIB-4 values might mainly result from improvement in necroinflammation.

**Electronic supplementary material:**

The online version of this article (10.1186/s12876-019-0973-5) contains supplementary material, which is available to authorized users.

## Background

Chronic hepatitis C (CHC) is a major health care concern worldwide. Histological staging of liver fibrosis is essential for treatment decision-making and prognostication in patients with CHC [1]. Owing to the invasive nature of liver biopsy, many noninvasive fibrosis indices have been developed to assess the stage of liver fibrosis [2]. Among these indices, the aspartate aminotransferase (AST)/platelet ratio index (APRI) [3] and FIB-4 index [4] are commonly used. These two noninvasive indices have been demonstrated to be reliable for predicting liver fibrosis in patients with CHC before therapy [5, 6].

Before the advent of direct-acting antiviral agents (DAAs), a combination therapy with pegylated interferon-α (Peg-IFN) and ribavirin (RBV) was the standard of care for patients with CHC [7, 8]. This combination therapy significantly reduced hepatic necroinflammation and liver fibrosis progression in patients with CHC [9]. However, some patients with CHC exhibited a persistent increase in alanine aminotransferase (ALT) level during Peg-IFN and RBV therapy [10]. The combination therapy also reduced the platelet count because of bone marrow suppression [11, 12]. Therefore, dynamic changes occurring in noninvasive index values cannot be accurately assessed and remain largely unknown in patients with CHC who receive Peg-IFN and RBV therapy.

The APRI and FIB-4 index have been used to evaluate fibrosis regression after antiviral therapy [13, 14]; however, Kim et al. reported that these two indices were not suitable for evaluating improvement of liver fibrosis in patients with chronic hepatitis B after antiviral therapy [15]. DAA therapy is currently the standard of care for patients with CHC [8, 16]. However, few studies have examined the dynamic changes occurring in the noninvasive index values and liver stiffness measurements (LSMs) of patients with CHC who have been treated with DAAs [17–19]. The present study investigated the temporal effect of DAAs on the noninvasive index values of patients with CHC at baseline, week 2, week 4, end of therapy (EOT), and 12 weeks after therapy (PW12) and identified factors associated with these changes.

## Methods

### Patients

Patients with CHC who received a complete course of DAA therapy from September 2012 to December 2017 were enrolled in this retrospective analysis. Inclusion criteria were as follows: age ≥ 18 years, presence of the serum anti-hepatitis C virus (HCV) antibody for > 6 months and detectable HCV RNA (detection limit = 15 IU/mL; COBAS Ampliprep/COBAS TaqMan HCV test, Roche Diagnostics, Branchburg, NJ, USA), and completion of DAA therapy. Exclusion criteria were as follows: presence of liver disease caused by other etiologies, decompensated liver disease, hepatocellular carcinoma at baseline, comorbid diseases or cancer, and concurrent use of eltrombopag or immunomodulatory agents. Demographic data, complete blood count analysis results, and biochemical data were collected at baseline, week 2, week 4, EOT, and PW12. Paired LSMs were performed using acoustic radiation force impulse elastography (ARFI) at baseline and PW12 in a subgroup of patients (*n* = 199). This study was conducted in accordance with the 1975 Declaration of Helsinki. All patients provided written informed consent prior to enrollment, and this study was approved by the Research Ethics Committee of China Medical University Hospital, Taichung, Taiwan (CMUH106-REC2–105).

### Laboratory tests

Complete blood count analyses (Sysmex HST-series, Sysmex, Kanogawa, Japan) and blood biochemistry tests (Beckman Coulter, Brea, CA, USA), including AST and ALT evaluations, were performed in the central laboratory of the hospital. HCV RNA was monitored at baseline, week 2, week 4, EOT, and PW12. HCV genotyping was performed using the Abbott RealTime HCV Genotype II assay (Abbott Molecular, Abbott Park, IL, USA). LSMs were performed using ARFI according to the process described in another study [20]. Reliable LSMs were defined as those having an interquartile range of < 30% of the median of 10 successful LSMs and a successful detection rate of > 60%. Liver cirrhosis was diagnosed by performing an unequivocal clinical, ultrasonographic, or pathological analysis.

### Noninvasive indices for liver fibrosis

We computed APRI [3] and FIB-4 values [4] using the following formulae:

$$ \mathrm{APRI}=\frac{\mathrm{AST}\left(/\mathrm{ULN}\right)}{\mathrm{Platelet}\ \mathrm{count}\ \left({10}^9/\mathrm{L}\right)}\times 100 $$

where ULN is the upper limit of normal and ULN = 34 U/L for AST.

$$ \left(\mathrm{FIB}\hbox{-} 4\right)=\frac{\mathrm{Age}\ \left(\mathrm{years}\right)\times \mathrm{AST}\ \left(\mathrm{U}/\mathrm{L}\right)}{\mathrm{Platelet}\ \mathrm{count}\left({10}^9/\mathrm{L}\right)\times \sqrt{\mathrm{ALT}\left(\mathrm{U}/\mathrm{L}\right)}} $$

### Statistical analyses

The normality of AST level, ALT level, and platelet count was assessed using the Kolmogorov–Smirnov test, which revealed a nonnormal distribution for these three parameters. The medians for continuous variables (first quartile–third quartile) were compared between the two groups and at different time points in the same group using the Mann–Whitney *U* test and the Wilcoxon signed-rank test, respectively. A two-sided *P* value of < 0.05 was considered statistically significant.

## Results

### Baseline characteristics

A total of 395 consecutive patients were enrolled retrospectively; their median age was 60 (52–67) years, and 179 (45.3%) of them were men. The baseline median AST, ALT, and total bilirubin levels were 54 (36–89) U/L, 65 (40–103) U/L, and 0.9 (0.6–1.2) mg/dL, respectively. The median platelet count was 142 (97–190) ×  10^9^/L. Furthermore, 133 (33.7%) patients had liver cirrhosis. In total, 326 (82.5%), 55 (13.9%), 1 (0.3%), 1 (0.3%), and 12 (3.0%) patients received diagnoses of HCV genotype (GT) infections 1, 2, 3, 4, and 6, respectively. The median HCV RNA level was 6.62 (6.08–7.09) log_10_ IU/mL, and the sustained virologic response (SVR) rate at 12 weeks after therapy (SVR12) was 98.2%. The median APRI value was 1.19 (0.62–2.45), and the median FIB-4 value was 2.93 (1.57–5.80). The median LSM obtained using ARFI was 1.73 (1.24–2.25) m/s (*n* = 252; Table 1). Regimens for the seven patients without SVR12 were sofosbuvir plus RBV for 12 weeks in two patients (GT 2a and GT 2b each), daclatasvir plus asunaprevir for 24 weeks in four patients (all GT 1b), and grazoprevir plus elbasvir for 12 weeks in one patient (GT 4). The regimens used and their treatment durations are listed in Additional file 1: Table S1. Furthermore, 85 (21.5%) patients received regimens containing RBV.

**Table 1:** Patient demographics and baseline characteristics

Status (*n* = 395)	*n* (%) or median (IQR)
Age (years)	60 (52–67)
Sex, M/F (% male)	179/216 (45.3)
Hemoglobin (g/dL)	13.9 (12.9–14.9)
Total bilirubin (mg/dL)	0.9 (0.6–1.2)
AST (U/L)	54 (36–89)
ALT (U/L)	65 (40–103)
Platelet count (× 10^9^/L)	142 (97–190)
HCV genotype, *n* (%)	
1	326 (82.5)
2	55 (13.9)
3	1 (0.3)
4	1 (0.3)
6	12 (3.0)
SVR, *n* (%)	388 (98.2)
Liver cirrhosis, *n* (%)	133 (33.7)
HCV RNA (log_10_ IU/mL)	6.62 (6.08–7.09)
APRI	1.19 (0.62–2.45)
FIB-4	2.93 (1.57–5.80)
LSM using ARFI (m/s)	1.73 (1.24–2.25) (*n* = 252)

*ALT* alanine aminotransferase, *APRI* AST/platelet ratio index, *AST* aspartate aminotransferase, *HCV* hepatitis C virus, *IQR* interquartile range, *LSM using ARFI* liver stiffness measurement using acoustic radiation force impulse elastography, *SVR* sustained virologic response

### APRI and FIB-4 values at different time points in patients with and without SVR12

**Fig. 1:**
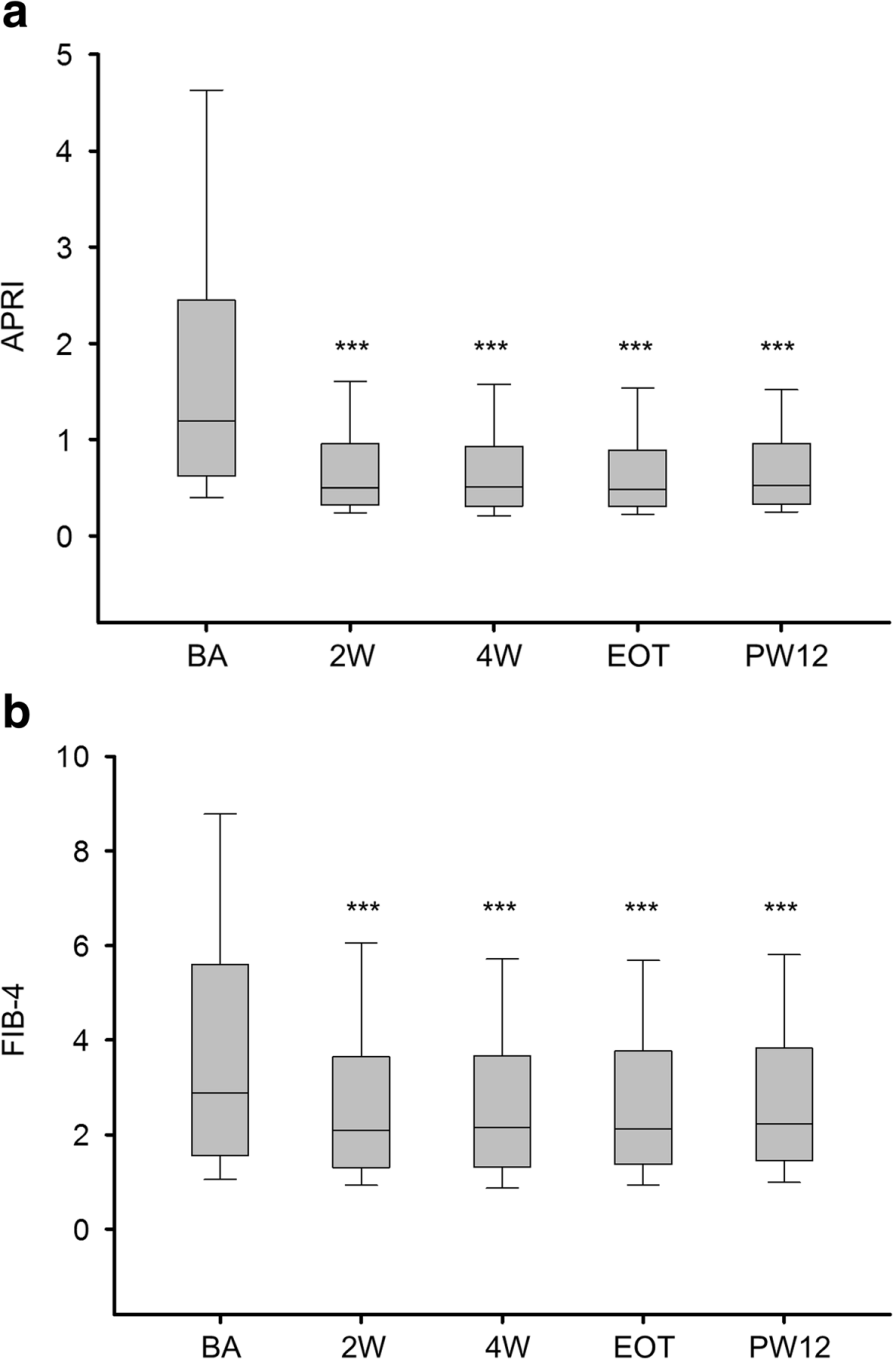
APRI and FIB-4 values at different time points in patients with SVR12 (*n* = 388). APRI (**a**). FIB-4 (**b**). APRI, AST/platelet ratio index; SVR12, sustained virologic response at 12 weeks after therapy; BA, baseline; 2 W, week 2; 4 W, week 4; EOT, end of therapy; PW12, 12 weeks after direct-acting antiviral therapy. All comparisons are made with baseline levels. ^***^P < 0.001

**Fig. 2:**
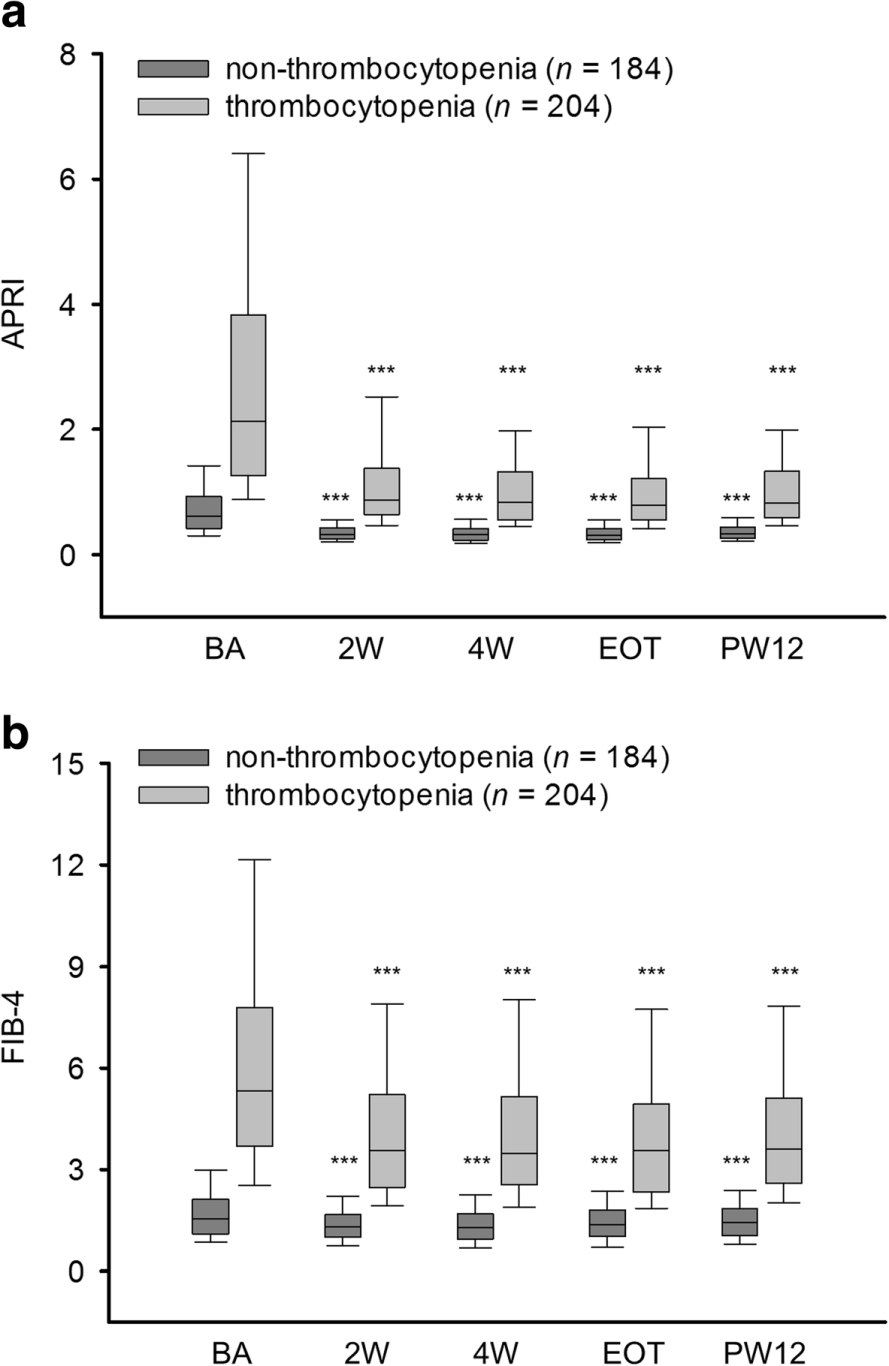
APRI and FIB-4 values at different time points in patients with (*n* = 204) and without (*n* = 184) thrombocytopenia who achieved SVR12. APRI (**a**). FIB-4 (**b**). APRI, AST/platelet ratio index; SVR12, sustained virologic response at 12 weeks after therapy; BA, baseline; 2 W, week 2; 4 W, week 4; EOT, end of therapy; PW12, 12 weeks after direct-acting antiviral therapy. All comparisons are made with baseline levels. ^***^P < 0.001

In patients who received DAA therapy and achieved SVR12 (*n* = 388), the median APRI and FIB-4 values decreased from week 2 until PW12 (Fig. 1a and b and Table 2). The corresponding AST and ALT levels and platelet count at different time points are listed in Table 2. Similar patterns for changes in APRI and FIB-4 values throughout the treatment and follow-up periods were observed in patients with (*n* = 204) and without (*n* = 184) thrombocytopenia (platelet count < 150 × 10^9^/L) at baseline, and the median APRI and FIB-4 values decreased from week 2 until PW12 (Fig. 2a and b and Table 2).

**Table 2:** Median AST, ALT, PLT, APRI, and FIB-4 values at different time points in different subgroups of patients with and without SVR12

Subgroups	Variables	Baseline	Week 2	Week 4	EOT	PW12
With SVR12						
All (*n* = 388)	AST (U/L)	55 (36–87)	27 (21–36)^***^	25 (20–35)^***^	25 (20–33)^***^	27 (21–34)^***^
	ALT (U/L)	66 (41–102)	25 (17–35)^***^	22 (16–33)^***^	21 (16–30)^***^	22 (16–29)^***^
	PLT (× 10^9^/L)	142 (99–190)	152 (108–202)^***^	163 (110–205)^***^	158 (106–202)^***^	151 (109–195)^***^
	APRI	1.19 (0.62–2.44)	0.50 (0.32–0.95)^***^	0.51 (0.31–0.92)^***^	0.48 (0.31–0.88)^***^	0.52 (0.33–0.92)^***^
	FIB-4	2.88 (1.56–5.60)	2.10 (1.30–3.65)^***^	2.15 (1.30–3.65)^***^	2.11 (1.37–3.76)^***^	2.22 (1.45–3.82)^***^
With thrombocytopenia (*n* = 204)	AST (U/L)	68 (46–113)	32 (25–41)^***^	31 (24–41)^***^	29 (23–37)^***^	31 (25–39)^***^
	ALT (U/L)	78 (48–123)	28 (21–41)^***^	25 (19–37)^***^	24 (18–34)^***^	25 (19–35)^***^
	PLT (× 10^9^/L)	100 (78–126)	112 (83–136)^***^	113 (87–137)^***^	110 (86–140)^***^	111 (84–131)^***^
	APRI	2.12 (1.26–3.83)	0.87 (0.64–1.38)^***^	0.79 (0.56–1.32)^***^	0.79 (0.55–1.21)^***^	0.83 (0.59–1.33)^***^
	FIB-4	5.34 (3.69–7.79)	3.58 (2.47–5.21)^***^	3.47 (2.55–5.15)^***^	3.56 (2.33–4.94)^***^	3.61 (2.59–5.12)^***^
Without thrombocytopenia (*n* = 184)	AST (U/L)	40 (30–60)	22 (19–28)^***^	21 (18–27)^***^	21 (18–27)^***^	22 (19–28)^***^
	ALT (U/L)	54 (35–81)	20 (16–29)^***^	18 (14–27)^***^	18 (14–27)^***^	18 (14–26)^***^
	PLT (× 10^9^/L)	192 (167–226)	204 (178–238)^**^	205 (177–241)^***^	206 (176–236)^***^	195 (171–226)
	APRI	0.61 (0.42–0.94)	0.32 (0.25–0.43)^***^	0.31 (0.23–0.42)^***^	0.31 (0.24–0.42)^***^	0.33 (0.26–0.44)^***^
	FIB-4	1.55 (1.10–2.13)	1.30 (1.00–1.66)^***^	1.30 (0.94–1.70)^***^	1.37 (1.02–1.81)^***^	1.44 (1.06–1.85)^***^
Without SVR12						
All (*n* = 7)	AST (U/L)	46 (34–217)	31 (19–59)^*^	29 (20–53)^*^	36 (19–55)^*^	40 (27–163)
	ALT (U/L)	58 (31–143)	28 (14–45)^*^	18 (14–39)^*^	31 (15–49)^*^	42 (16–153)
	PLT (× 10^9^/L)	87 (50–198)	99 (52–197)	130 (74–197)	95 (45–166)	99 (63–170)
	APRI	2.71 (0.44–7.47)	0.84 (0.27–2.62)^*^	0.82 (0.28–2.30)^*^	1.42 (0.33–2.91)^*^	1.87 (0.43–6.86)^*^
	FIB-4	5.92 (1.89–14.48)	4.20 (1.69–9.00)^*^	4.54 (1.74–8.71)	4.52 (2.16–9.94)	4.80 (2.36–14.05)

Data in the table presented as the median (first quartile–third quartile), and all comparisons made with baseline levels

*ALT* alanine aminotransferase, *APRI* aspartate aminotransferase/platelet ratio index, *AST* aspartate aminotransferase, *EOT* end of therapy, *PLT* platelet count, *PW12* 12 weeks after direct-acting antiviral therapy, *SVR12* sustained virologic response at 12 weeks after therapy

^*^P < 0.05, ^**^P < 0.01, ^***^P < 0.001

In patients without SVR12 (*n* = 7), the median APRI value significantly decreased from week 2 until PW12, and the magnitude of changes decreased at EOT and PW12 (Table 2 and Additional file 2: Figure S1a). The median FIB-4 value decreased at week 2 and then returned to counts similar to the baseline value from week 4 until PW12. The corresponding AST and ALT levels and platelet count at different time points are listed in Table 2 and Additional file 2: Figure S1b.

### Platelet count and ALT levels at different time points in patients with SVR12

**Fig. 3:**
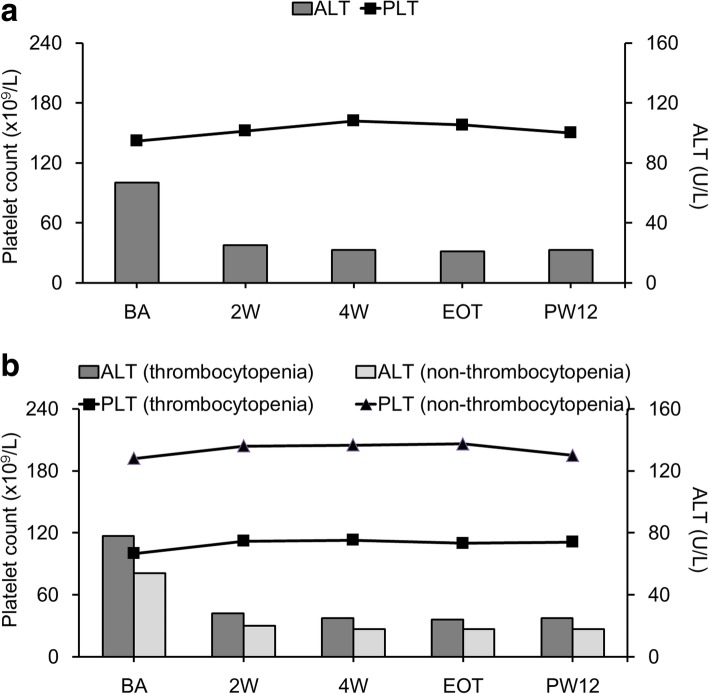
Platelet count and ALT levels at different time points in all patients with SVR12 (*n* = 388) (**a**), those with (*n* = 204) and without (*n* = 184) thrombocytopenia who achieved SVR12 (**b**). ALT, alanine aminotransferase; BA, baseline; 2 W, week 2; 4 W, week 4; EOT, end of therapy; PW12, 12 weeks after direct-acting antiviral therapy

In patients who received DAA therapy and achieved SVR12 (*n* = 388), the median platelet count increased from week 2 until PW12. The median platelet count at PW12 was significantly lower than the peak platelet count at week 4 (*P* < 0.001). The median ALT level decreased from week 2 until PW12 (Fig. 3a and Table 2).

In patients with thrombocytopenia at baseline (*n* = 204), the median platelet count increased from week 2 until PW12. The median platelet count at PW12 was similar to those at week 2, week 4, and EOT (all *P* > 0.05). The median ALT levels decreased from week 2 until PW12 (Fig. 3b and Table 2). Patients without thrombocytopenia showed similar patterns for changes in ALT level but different patterns for changes in platelet count (*n* = 184). The median platelet count increased from week 2 until EOT and returned to a count similar to the baseline value at PW12 (*P* = 0.764). The median ALT level decreased from week 2 until PW12 (Fig. 3b and Table 2).

In patients without SVR12 (*n* = 7), the median ALT levels decreased from week 2 until EOT and then returned to the baseline level at PW12 (*P* = 0.128). No significant differences were observed in the median platelet count at week 2, week 4, EOT, and PW12 compared with that at baseline (Table 2).

### LSMs obtained using ARFI at baseline and PW12

**Fig. 4:**
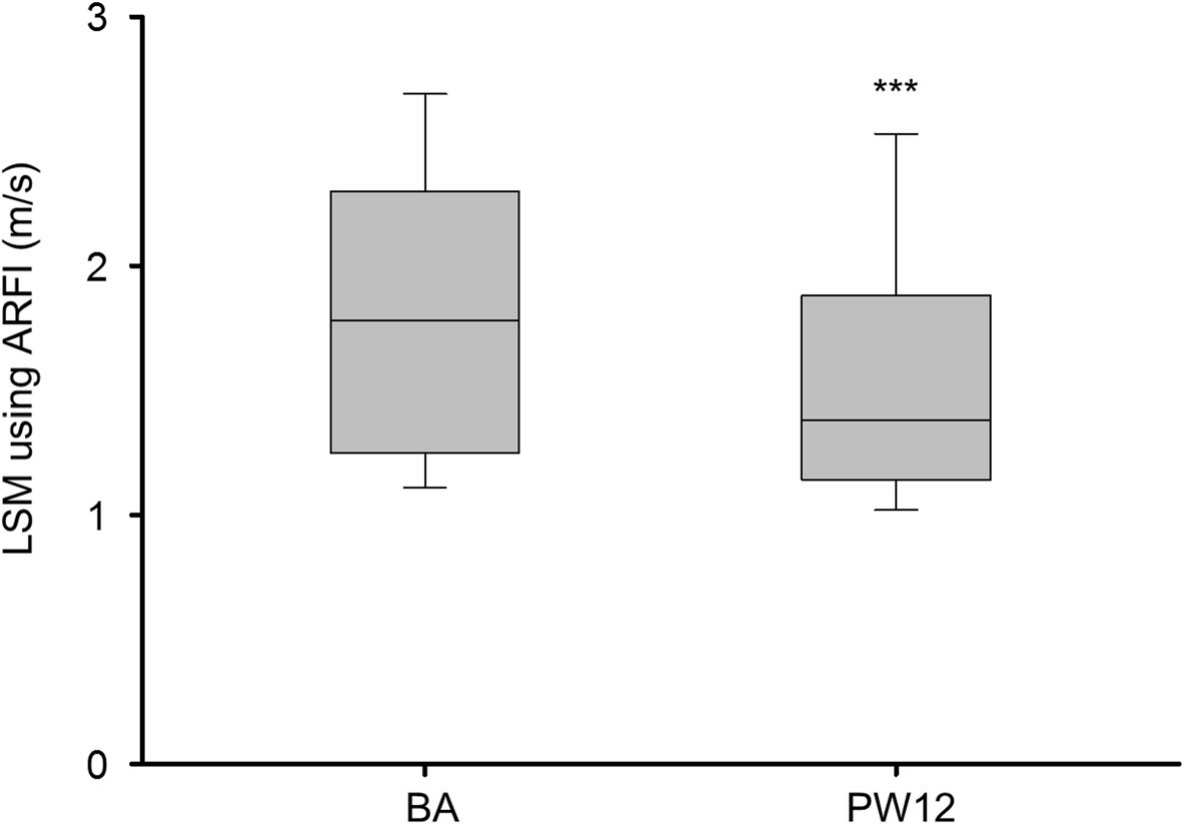
LSM obtained using ARFI in patients with SVR12 and paired measurements at baseline and PW12. LSM, liver stiffness measurement; ARFI, acoustic radiation force impulse elastography; SVR12, sustained virologic response at 12 weeks after therapy; BA, baseline; PW12, 12 weeks after direct-acting antiviral therapy. All comparisons are made with baseline levels. ^***^P < 0.001

In patients who received DAA therapy and achieved SVR12 with their paired LSMs obtained using ARFI at baseline and PW12 (*n* = 199), the median LSM decreased from 1.78 (1.25–2.30) m/s at baseline to 1.38 (1.14–1.88) m/s at PW12 (*P* < 0.001) (Fig. 4).

## Discussion

Values for noninvasive fibrosis indices, namely the APRI and FIB-4 index, rapidly decreased at 2 weeks after the initiation of DAA therapy in various subgroups of patients with CHC, and the median APRI and FIB-4 values decreased from week 2 until PW12 in patients who achieved SVR12. LSMs obtained using ARFI also significantly decreased at PW12, which is consistent with LSMs obtained using transient elastography (TE) in previous studies [17–19]. Studies have revealed regression of APRI and FIB-4 values at EOT [18] and PW12 [17, 18]. The results of the present study demonstrated that the decline in APRI and FIB-4 values occurred as early as 2 weeks after treatment initiation and remained sustained thereafter until PW12. The rapid regression of APRI and FIB-4 values might be due to the rapid decline in AST and ALT levels, and to a lesser extent, increased platelet count since 2 weeks after DAA therapy initiation.

Both the APRI and FIB-4 index exhibited a strong correlation with liver fibrosis stage before antiviral therapy [3–6]. Significant changes in APRI and FIB-4 values from baseline to 2 weeks after DAA therapy initiation may refute its causal relationship with fibrosis regression, which may take considerably longer to develop. Instead, these changes in APRI and FIB-4 values might mainly result from the rapid decline in AST and ALT levels due to improvement in necroinflammation. Similarly, Elsharkawy et al. demonstrated a significant reduction in AST and ALT levels and APRI values at week 4 and EOT in patients with CHC GT 4 who received sofosbuvir-based regimens. They suggested that early reduction in the APRI value mostly reflected reduction in necroinflammation rather than in fibrosis regression [21]. The increased median APRI and FIB-4 values at EOT and PW12 compared with those at week 2 were also correlated with temporal changes in AST and ALT levels in patients without SVR12 (Additional file 2: Figure S1a and S1b). Although there was a significant increase in the median platelet count from week 2 until EOT in all subgroups of patients and at PW12 in patients with thrombocytopenia, the magnitude of changes in platelet count was low (Fig. 3a and b).

Patients with CHC who achieved SVR subsequently developed fewer liver-related adverse events, such as liver cirrhosis, hepatic decompensation, and hepatocellular carcinoma, than did those without SVR [22]. However, SVR does not guarantee the elimination of liver-related adverse events, and post-SVR programmed surveillance is necessary [23]. The residual liver fibrosis stage is a crucial determinant of liver-related adverse events in patients who achieve SVR [24]. A systematic review showed that LSM performed using ARFI is an accurate and reliable method for staging liver fibrosis in treatment-naïve patients with CHC [25]; however, few studies have examined temporal changes in LSMs when using ARFI in patients with CHC who receive DAA therapy. In our previous study, we observed a significant decrease in LSM values obtained using ARFI in patients with CHC after antiviral therapy, including Peg-IFN plus RBV and DAA therapies [26]. The decline in LSMs obtained using ARFI or TE at 12 or 24 weeks after EOT raises the question of its underlying cause [19, 26–28]. Arena et al. demonstrated that hepatic necroinflammatory activity strongly and independently influences LSMs obtained using TE [29]. Singh et al. showed that LSMs obtained using TE decreased during the 6–12 months after viral eradication and concluded that early decline in liver stiffness was probably related to the resolution of necroinflammation [30]. Therefore, LSM during the period of necroinflammatory resolution may not be optimal for staging fibrosis.

Although rapid reductions in APRI values, FIB-4 values, and LSMs obtained using ARFI may mainly result from improvement in hepatic necroinflammation rather than fibrosis regression, temporal changes in the noninvasive index can be a predictor of fibrosis regression in patients with CHC who receive Peg-IFN-based therapy. Tanwar et al. demonstrated that a change in the enhanced liver fibrosis test (hyaluronic acid, terminal peptide of procollagen III, and tissue inhibitor of matrix metaloproteinase-1) from baseline to 12 months after Peg-IFN-based therapy initiation, in combination with baseline histologic staging, could predict histologic fibrosis regression at 24 months after the therapy [31]. Nonetheless, the results of the present study suggest that noninvasive index values obtained during or soon after DAA therapy may not be predictive of the concomitant fibrosis stage, and an additional histology-based correlative study is required to establish optimal cutoffs for predicting fibrosis stages.

Common factors affecting platelet count in patients with CHC are liver fibrosis, hepatic necroinflammation [32], and thrombopoietin [33]. Karasu et al. demonstrated that liver fibrosis stage was inversely associated with platelet count in patients with chronic hepatitis B and CHC [32]. Platelets interact with the hepatic sinusoidal endothelium while circulating in the injured liver and recruit effector cells and proteins [34]. This activity causes a self-perpetuating cycle of platelet and leukocyte accumulation, resulting in hepatocellular injury [35]. One study demonstrated that hepatic necroinflammatory activity was correlated to a low platelet count in patients with CHC [36]. However, the effect of changes in ALT level on platelet count during Peg-IFN and RBV therapy could not be assessed due to bone marrow suppression by Peg-IFN [11, 12]. Our study demonstrated that reduction in hepatic necroinflammation, as evidenced by decreased ALT levels, was correlated chronologically with an increased platelet count at week 2, week 4, EOT, and PW12 in various subgroups of patients. This suggests that the platelet count increased as a result of reduced necroinflammation. Deterding et al. and Pons et al. both reported increased platelet count early during treatment until 12 and 24 weeks after DAA therapy. However, these studies enrolled few patients (*n* = 80 and 41, respectively) and did not investigate dynamic changes in the platelet count of patients without thrombocytopenia [37, 38]. Giannini et al. demonstrated that a progressive decline in liver function in patients with HCV-related chronic liver disease was correlated with a decrease in thrombopoietin production [39]. Increased thrombopoietin production following reduction in necroinflammation may have been partially responsible for the increased platelet count observed in this study. Furthermore, in patients with available data on platelet count and ALT level at week 1, 213 out of 224 (95.1%) patients exhibited a decline in ALT of > 5%, whereas 110 of 213 (51.6%) patients exhibited an increase of > 5% in platelet count. This observation supports the hypothesis that the platelet count increased later as a consequence of reduction in necroinflammation. However, the present study could not clearly define the time interval between these two events.

This study has several limitations. First, a histological study was not conducted to examine correlations with temporal changes occurring in noninvasive fibrosis indices during and after DAA therapy. However, liver biopsy cannot be widely applied in clinical practice because of increasing patient reluctance to undergo this invasive procedure. Second, this study was conducted at a single center, and only 395 patients were enrolled. Third, 11 regimens were used, and investigating temporal changes in APRI values, FIB-4 values, and LSMs for a single regimen was difficult because of the limited number of patients who were administered each regimen. Future studies involving the same DAA regimen are warranted. Fourth, LSM was not performed along with these indices during DAA therapy, which required a prospective study design. Finally, laboratory data were collected only until PW12. Consequently, long-term changes in APRI and FIB-4 values, related biochemical parameters, and LSMs require further investigation. Despite these limitations, the present study demonstrated a rapid decline in APRI and FIB-4 values from 2 weeks after DAA therapy initiation until 12 weeks after DAA therapy, thereby highlighting a pitfall of monitoring fibrosis status using APRI and FIB-4 values in patients with CHC who receive DAA therapy.

## Conclusions

Noninvasive fibrosis indices, namely APRI and FIB-4, exhibited a rapid and sustained decline from week 2 until PW12 in patients with CHC who achieved SVR to DAA therapy. The rapid decline in APRI and FIB-4 values might primarily result from reduction in necroinflammation. Additional studies are warranted to elucidate the relationship between a noninvasive index and fibrosis stage in post-SVR patients with CHC.

## Additional files

Additional file 1: **Table S1.** Regimens used in this study. (DOCX 16 kb)

Additional file 2: **Figure S1.** APRI and FIB-4 values at different time points in patients without SVR12 (*n* = 7). APRI (**a**). FIB-4 (**b**). APRI, AST/platelet ratio index; SVR12, sustained virologic response at 12 weeks after therapy; BA, baseline; 2W, week 2; 4W, week 4; EOT, end of therapy; PW12, 12 weeks after direct-acting antiviral therapy. All comparisons are made with baseline levels. ^*^P < 0.05. (ZIP 83 kb)

## Abbreviations

ALT: Alanine aminotransferase
APRI: Aspartate aminotransferase/platelet ratio index
ARFI: Acoustic radiation force impulse elastography
AST: Aspartate aminotransferase
CHC: Chronic hepatitis C
DAAs: Direct-acting antiviral agents
EOT: End of therapy
GT: Genotype
HCV: Hepatitis C virus
LSM: Liver stiffness measurement
Peg-IFN: Pegylated interferon-α
PW12: 12 weeks after therapy
RBV: Ribavirin
SVR: Sustained virologic response
SVR12: SVR at 12 weeks after therapy
TE: Transient elastography
ULN: Upper limit of normal
