# Supplementary material for: Rapid decline of noninvasive fibrosis index values in patients with hepatitis C receiving treatment with direct-acting antiviral agents
Source: BMC Gastroenterol. 2019 Apr 27;19:63. doi: 10.1186/s12876-019-0973-5 (PMC6486982; doi:10.1186/s12876-019-0973-5)
Supplement: Supplementary file 1 — Table S1. Regimens used in this study. (DOCX 16 kb) [file 12876_2019_973_MOESM1_ESM.docx]

**Additional file 1**

**Table S1** Regimens used in this study

| Regimens (*n* = 395) | Duration (weeks) | *n* (%) |
| --- | --- | --- |
| GZR + EBR | 12–16 | 69 (17.5) |
| GZR + EBR + RBV | 12–16 | 10 (2.5) |
| DCV + ASV | 24 | 71 (18.0) |
| SOF + LDV | 12 | 36 (9.1) |
| SOF + LDV + RBV | 12 | 31 (7.8) |
| SOF + DCV | 12 | 8 (2.0) |
| SOF + VEL | 12 | 7 (1.8) |
| SOF + VEL + RBV | 12 | 2 (0.5) |
| SOF + RBV | 12 | 32 (8.1) |
| PrOD | 12 | 119 (30.1) |
| PrOD + RBV | 12 | 10 (2.5) |
|  |  |  |
| Regimens including RBV | 12–16 | 85 (21.5) |

*ASV* asunaprevir, *DCV* daclatasvir, *EBR* elbasvir, *GZR* grazoprevir, *LDV* ledipasvir, *PrOD* paritaprevir/ritonavir/ombitasvir plus dasabuvir, *RBV* ribavirin, *SOF* Sofosbuvir, *VEL* velpatasvir
