# Supplementary figures and images for: Rapid decline of noninvasive fibrosis index values in patients with hepatitis C receiving treatment with direct-acting antiviral agents
Source: BMC Gastroenterol. 2019 Apr 27;19:63. doi: 10.1186/s12876-019-0973-5 (PMC6486982; doi:10.1186/s12876-019-0973-5)

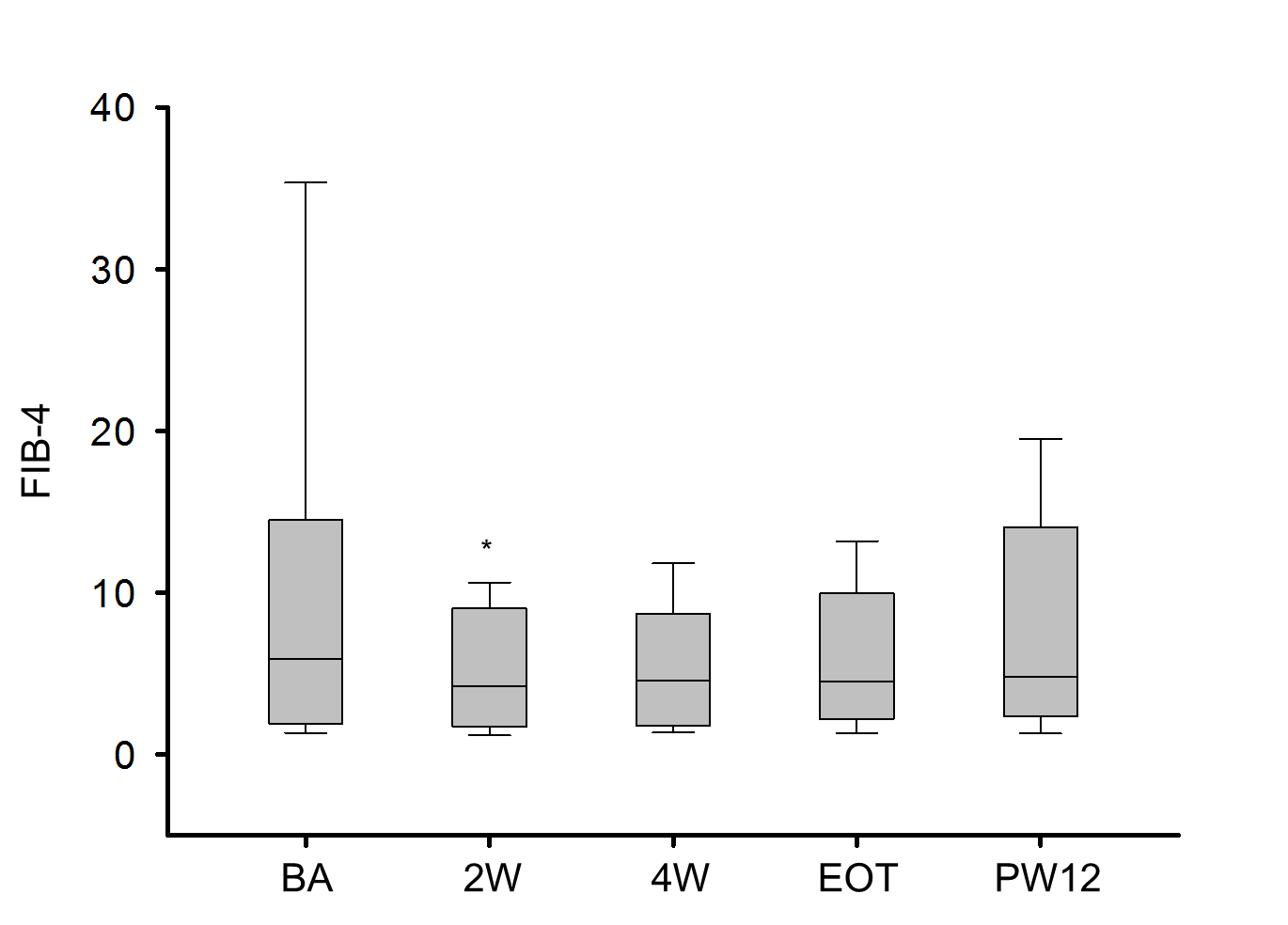

Supplement: Supplementary file 2 — Figure S1. APRI and FIB-4 values at different time points in patients without SVR12 (n = 7). APRI (a). FIB-4 (b). APRI, AST/platelet ratio index; SVR12, sustained virologic response at 12 weeks after therapy; BA, baseline; 2W, week 2; 4W, week 4; EOT, end of therapy; PW12, 12 weeks after direct-acting antiviral therapy. All comparisons are made with baseline levels. *P < 0.05. (ZIP 83 kb) [file 12876_2019_973_MOESM2_ESM.zip › Fig. S1bR1.tif]

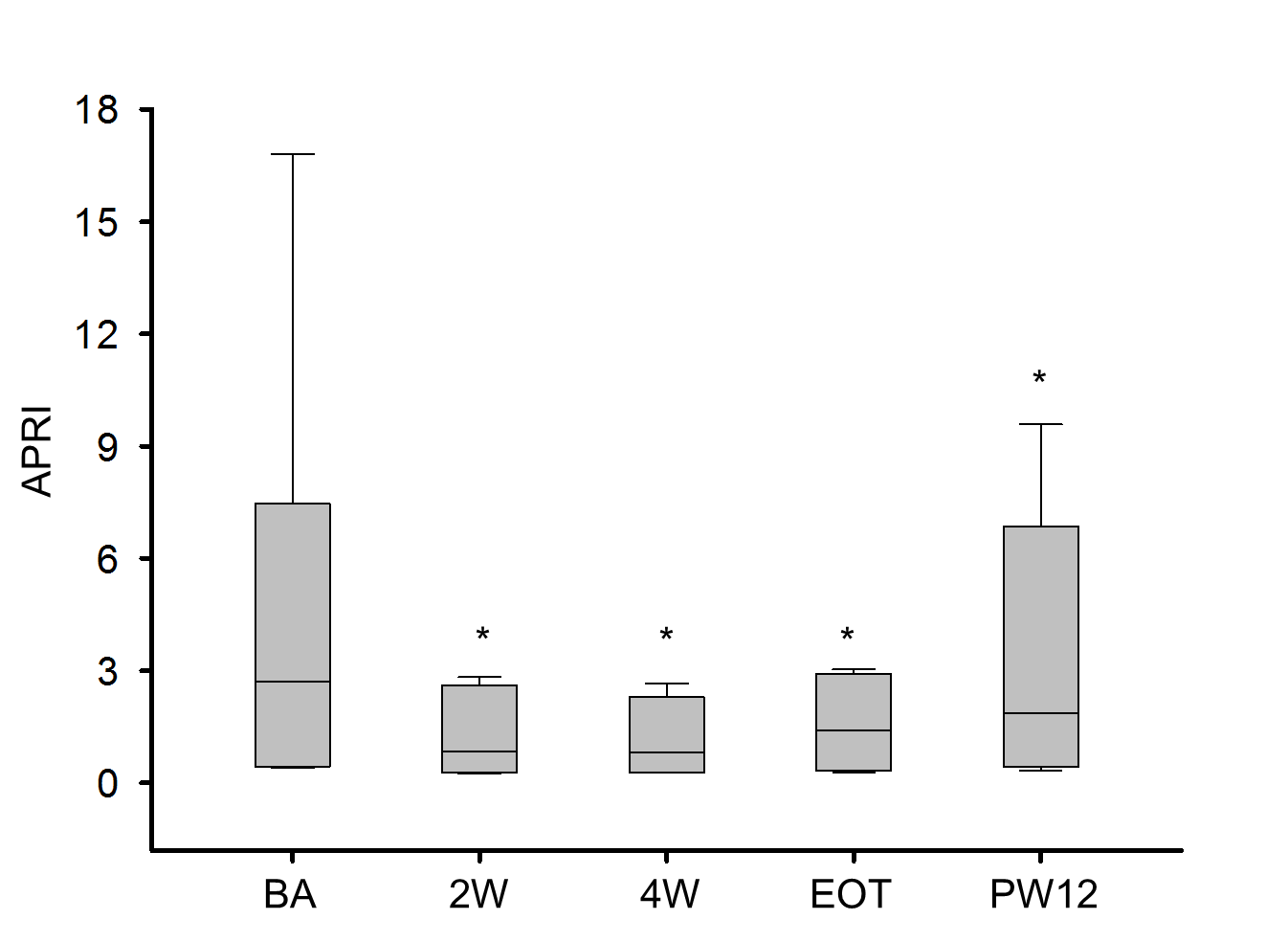

Supplement: Supplementary file 2 — Figure S1. APRI and FIB-4 values at different time points in patients without SVR12 (n = 7). APRI (a). FIB-4 (b). APRI, AST/platelet ratio index; SVR12, sustained virologic response at 12 weeks after therapy; BA, baseline; 2W, week 2; 4W, week 4; EOT, end of therapy; PW12, 12 weeks after direct-acting antiviral therapy. All comparisons are made with baseline levels. *P < 0.05. (ZIP 83 kb) [file 12876_2019_973_MOESM2_ESM.zip › Fig. S1aR1.tif]
